# Minocycline modulates antigen-specific CTL activity through inactivation of mononuclear phagocytes in patients with HTLV-I associated neurologic disease

**DOI:** 10.1186/1742-4690-9-16

**Published:** 2012-02-15

**Authors:** Yoshimi Enose-Akahata, Eiji Matsuura, Yuetsu Tanaka, Unsong Oh, Steven Jacobson

**Affiliations:** 1 Viral Immunology Section, Neuroimmunology Branch, National Institute of Neurological Disorders and Stroke, National Institutes of Health, Bethesda, Maryland 20892 USA; 2 Department of Neurology and Geriatrics, Graduate School of Medical and Dental Sciences, Kagoshima University, Kagoshima 890-8544 Japan; 3 Department of Immunology, Graduate School and Faculty of Medicine, University of the Ryukyus, Okinawa 903-0215 Japan; 4 Department of Neurology, Virginia Commonwealth University School of Medicine, Richmond, Virginia 23298 USA

**Keywords:** HTLV-I, HAM/TSP, monocyte, CTL, minocycline

## Abstract

**Background:**

The activation of mononuclear phagocytes (MPs), including monocytes, macrophages and dendritic cells, contributes to central nervous system inflammation in various neurological diseases. In HTLV-I-associated myelopathy/tropical spastic paraparesis (HAM/TSP), MPs are reservoirs of HTLV-I, and induce proinflammatory cytokines and excess T cell responses. The virus-infected or activated MPs may play a role in immuneregulation and disease progression in patients with HTLV-I-associated neurological diseases.

**Results:**

Phenotypic analysis of CD14^+ ^monocytes in HAM/TSP patients demonstrated high expression of CX_3_CR1 and HLA-DR in CD14^low^CD16^+ ^monocytes, compared to healthy normal donors (NDs) and asymptomatic carriers (ACs), and the production of TNF-α and IL-1β in cultured CD14^+ ^cells of HAM/TSP patients. CD14^+ ^cells of HAM/TSP patients also showed acceleration of HTLV-I Tax expression in CD4^+ ^T cells. Minocycline, an inhibitor of activated MPs, decreased TNF-α expression in CD14^+ ^cells and IL-1β release in PBMCs of HAM/TSP patients. Minocycline significantly inhibited spontaneous lymphoproliferation and degranulation/IFN-γ expression in CD8^+ ^T cells of HAM/TSP patients. Treatment of minocycline also inhibited IFN-γ expression in CD8^+ ^T cells of HAM/TSP patients after Tax11-19 stimulation and downregulated MHC class I expression in CD14^+ ^cells.

**Conclusion:**

These results demonstrate that minocycline directly inhibits the activated MPs and that the downregulation of MP function can modulate CD8^+ ^T cells function in HAM/TSP patients. It is suggested that activated MPs may be a therapeutic target for clinical intervention in HAM/TSP.

## Background

The human T cell lymphotropic virus I (HTLV-I) infects 20 million people worldwide of which the majority of infected individuals are asymptomatic carriers (AC) of the virus [1]. However, in a small percentage of infected individuals, HTLV-I is the etiologic agent of adult T cell leukemia/lymphoma (ATL) [2] and a chronic, progressive neurological disease termed HTLV-I-associated myelopathy/tropical spastic paraparesis (HAM/TSP) [3,4]. Patients with HAM/TSP demonstrate high HTLV-I proviral DNA load, high HTLV-I Tax mRNA load, and high virus-specific immune responses, including increased production of inflammatory cytokines and expansion of Tax-specific CD8^+ ^T cells [5-9]. A high frequency of CD4^+ ^T cells is persistently infected and exhibits high expression of Tax protein [10]. These infected cells are responsible for the increased lymphocyte proliferation in patients with HAM/TSP [11]. High frequency of activated CD8^+ ^T cells in peripheral blood and even higher in cerebrospinal fluid has been reported [12]. In addition to these strong HTLV-I-associated T cell responses, it has been suggested that mononuclear phagocytes (MPs; monocytes, dendritic cells, tissue macrophages and microglia) are also involved in the pathogenesis of HAM/TSP. MPs are infected with HTLV-I *in vitro *and *in vivo *[13-18], and dendritic cells have been shown to effectively transfer cell-free virus to CD4^+ ^T cells [18]. HTLV-I-infected dendritic cells can stimulate both CD4^+ ^and CD8^+ ^T cells [17]. Moreover, HTLV-I infection of CD14^+ ^cells and the concomitant expression of IL-15 mediate spontaneous degranulation and IFN-γ expression in CD8^+ ^T cells [19]. Pathological studies have confirmed the presence of inflammatory monocyte/macrophages as well as CD4^+ ^T cells and CD8^+ ^T cells in the central nervous system (CNS) of HAM/TAP patients [20,21]. These findings suggest that virus-infected or activated MPs may play a role in immune regulation and disease progression in patients with HTLV-I-associated neurological diseases.

MPs are widely distributed immune cells that maintain tissue homeostasis and provide a first line of defense against invading pathogens. MPs have been shown to present antigens bound by major histocompatibility complex (MHC) molecules and to activate CD4^+ ^T helper cells or cytotoxic CD8^+ ^T cells [22]. The abilities to combat microbial infection and clear debris are intimately tied to MP activation and follow degenerative, inflammatory, infectious, and ischemic insults. However, under inflammatory conditions, differential MP population and activation of MPs are related to immunopathogenesis and disease progression. Human peripheral monocytes contain two major subsets, the CD14^+^CD16^- ^and CD14^low^CD16^+ ^monocytes [23]. The CD14^low^CD16^+ ^monocytes express higher levels of proinflammatory cytokines than CD14^+^CD16^- ^monocytes, with a higher capacity for antigen presentation, and are increased in inflammatory and infectious diseases in humans [24]. Macrophage/microglial inflammatory activities have been shown to influence a number of neurodegenerative diseases including human immunodeficiency virus (HIV)-associated dementia, Alzheimer's disease, Parkinson's disease, stroke, brain and spinal cord trauma [25]. In HAM/TSP, the expression of proinflammatory cytokines such as IL-1β, TNF-α and IFN-γ is detected in peripheral blood mononuclear cells (PBMCs) as well as in perivascular infiltrating macrophages and microglia in the spinal cords of patients with HAM/TSP [26,27]. Moreover, HTLV-I Tax has been reported to induce the human proIL-1β gene promoter in monocytic cells [28]. Thus, MPs of patients with HAM/TSP might be activated under inflammatory conditions and play a role in immunopathogenesis of this disorder.

In this study, we demonstrate that CD14^+ ^cells of patients with HAM/TSP showed an inflammatory phenotype as evidenced by high expression of HLA-DR and CX_3_CR1, proinflammatory cytokines (TNF-α and IL-1β) and acceleration of HTLV-I Tax expression in CD4^+ ^T cells. Minocycline, which is tetracycline derivative and a known inhibitor of activated macrophage/microglia [29], significantly inhibited TNF-α and IL-1β expressions in cultured CD14^+ ^cells of patients with HAM/TSP. Moreover, treatment with minocycline demonstrated inhibition of IFN-γ expression in CD8^+ ^T cells of patients with HAM/TSP, resulting from inhibition of MP activation by minocycline. These results demonstrate that CD8^+ ^T cell activation of patients with HAM/TSP can be suppressed through down-regulation of MP activation, and suggest a novel treatment strategy in patients with HTLV-I associated neurological disease.

## Results

### High CX_3_CR1 and HLA-DR expression in monocytes of patients with HAM/TSP

**Figure 1:**
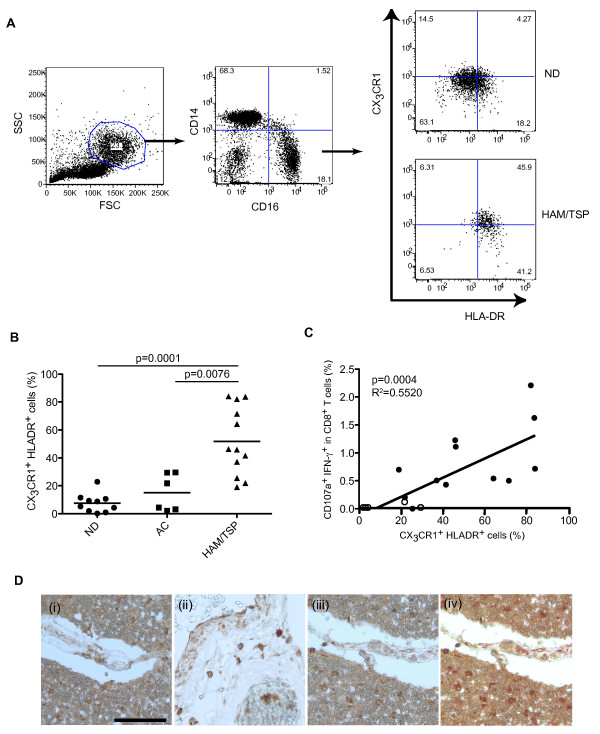
**Characterization of mononuclear phagocytes in patients with HAM/TSP**. (A) Representative dot plots of CX_3_CR1 and HLA-DR expression in CD14^low ^CD16^+ ^cells of a ND and a HAM/TSP patient. (B) Comparison of frequencies of CX_3_CR1^+ ^HLA-DR^+ ^cells in CD14^low ^CD16^+ ^mononuclear phagocytes of NDs, ACs and HAM/TSP patients. The data were obtained from ten NDs, six ACs and twelve HAM/TSP patients. The CD14^low^CD16^+ ^subset of HAM/TSP patients had significantly higher levels of both CX_3_CR1 and HLA-DR expression, compared to NDs (p = 0.0001) and ACs (p = 0.0076) by Mann-Whitney test. The horizontal line represents the mean. (C) The frequency of CX_3_CR1^+^HLA-DR^+ ^cells was shown to be significantly correlated with spontaneous degranulation/IFN-γ expressions in CD8^+ ^T cells of HTLV-I-infected patients, including ACs (n = 6, opened circle) and patients with HAM/TSP (n = 12, closed circle) by simple linear regression analysis (P = 0.0004, R^2 ^= 0.5520). (D) Localization of CX_3_CR1^+ ^cells in the spinal cord of a HAM/TSP patient. Parenchyma (i) and meninges (ii) were stained with antibodies for CX_3_CR1 (brown). Parenchyma was stained with antibody for CX_3_CR1 (brown in iii), and double-stained with CX_3_CR1 and CD68 (red in iv). CX_3_CR1^+ ^cells were positive for CD68. Magnifications, ×20. Black bar = 40 μm.

To characterize CD14^+ ^cell subsets in PBMCs of HAM/TSP patients, the expression of monocyte markers CD14 and CD16 was examined by flow cytometry in NDs, ACs and patients with HAM/TSP. Figure 1A demonstrates a representative dot plot of MP populations of a ND and a patient with HAM/TSP. Group analysis did not show significant differences between CD14^+^CD16^- ^and CD14^low^CD16^+ ^frequencies in MP population among NDs, ACs, and patients with HAM/TSP (data not shown). Previous reports demonstrated that CD14^low^CD16^+ ^monocytes expressed higher levels of CX_3_CR1 (a fractalkine receptor) and HLA-DR, proinflammatory cytokines and higher potency in antigen presentation in human inflammatory and infectious diseases [23,24]. We therefore compared CX_3_CR1 and HLA-DR expression on CD14^low^CD16^+ ^monocytes among the groups. A representative dot plot shows that both CX_3_CR1 and HLA-DR expression was higher in CD14^low^CD16^+ ^subset of a patient with HAM/TSP than that of a ND (Figure 1A). In NDs, the CD14^low^CD16^+ ^subset expressed both CX_3_CR1 and HLA-DR (mean+/-standard deviation (SD) = 7.572+/-6.748, n = 10; Figure 1B). In contrast, the CD14^low^CD16^+ ^subset of patients with HAM/TSP had significantly higher levels of both CX_3_CR1 and HLA-DR expression (mean+/-SD = 51.88+/-24.42, n = 12; Figure 1B). CX_3_CR1 and HLA-DR expression in CD14^low^CD16^+ ^subset of ACs was significantly lower than those in patients with HAM/TSP, and at comparable levels with those in NDs (mean+/-SD = 15.04+/-13.31, n = 6; Figure 1B). These results demonstrated that the CD14^low^CD16^+ ^subset in patients with HAM/TSP showed significantly high expression of CX_3_CR1 and HLA-DR, compared to NDs and ACs.

Given the high expression of CX_3_CR1 and HLA-DR on the CD14^low^CD16^+ ^subset in patients with HAM/TSP, we asked whether these changes in MP subsets were related to biomarkers of disease activity in HAM/TSP. We previously reported that CD14^+ ^cells induced degranulation and IFN-γ expression in CD8^+ ^T cells of patients with HAM/TSP *in vitro *[19]. We therefore analyzed the relationship between CX_3_CR1/HLA-DR expression on CD14^low^CD16^+ ^subset and degranulation/IFN-γ expression in CD8^+ ^T cells of HTLV-I-infected patients. CX_3_CR1/HLA-DR expression on CD14^low^CD16^+ ^subset was significantly correlated with degranulation/IFN-γ expression in CD8^+ ^T cells of HTLV-I-infected patients (Figure 1C; P = 0.0004, R^2 ^= 0.552). These results suggested that activation of MP *in vivo *could be related to CD8^+ ^T cell activation of patients with neurologic inflammatory disease.

Immunohistochemical analysis further demonstrated that CX_3_CR1^+ ^cells were detected in the spinal cord of a patient with HAM/TSP (Figure 1D). CX_3_CR1^+ ^cells were detected around the blood vessels and in the parenchyma and the meninges in the HAM/TSP spinal cord (Figure 1Di and 1Dii, respectively), suggesting a recruitment of CX_3_CR1^+ ^cells from the periphery to the spinal cord parenchyma and meninges. Moreover, CX_3_CR1^+ ^cells in the parenchyma were morphologically bigger (Figure 1Diii) and positive for CD68 (Figure 1Div), probably corresponding to MPs. These results further support the idea that CX_3_CR1^+ ^cells might be recruited from peripheral blood to the spinal cord in patients with HAM/TSP.

### CD14^+ ^cells express TNF-α and IL-1β and increase HTLV-I Tax expression in CD4^+ ^T cells of patients with HAM/TSP

**Figure 2:**
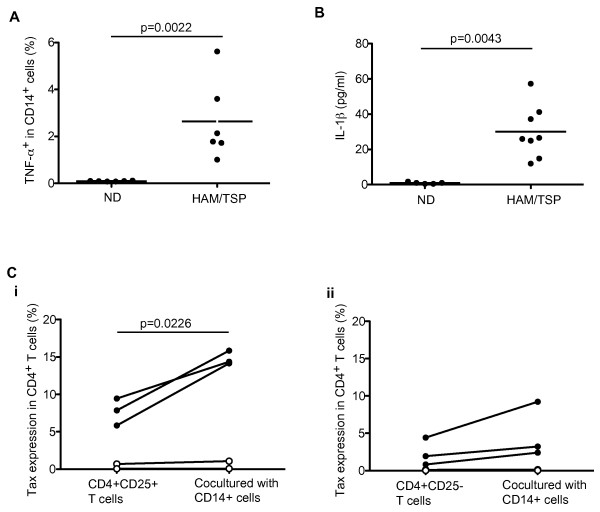
**Activated CD14^+ ^cells in patients with HAM/TSP**. (A) TNF-α expression in CD14^+ ^cells of NDs and HAM/TSP patients after culture for 24 hours. The data were obtained from six NDs and six HAM/TSP patients. CD14^+ ^cells expressing TNF-α was significantly elevated in HAM/TSP patients, compared to NDs by Mann-Whitney test (p = 0.0022). The horizontal line represents the mean. (B) Detection of IL-1β in PBMC culture supernatants of NDs and HAM/TSP patients after culture for 24 hours. The data were obtained from five NDs and eight HAM/TSP patients. IL-1β expression in HAM/TSP patients was significantly higher in those cells of NDs by Mann-Whitney test (p = 0.0043). The horizontal line represents the mean. (C) Tax expressions in CD4^+^CD25^+ ^T cells (i) and CD4^+^CD25^- ^T cells (ii) cocultured with or without autologous CD14^+ ^cells of ACs (n = 2, opened circle) and patients with HAM/TSP (n = 3, closed circle) for 18 hours.

To further investigate MP activation in HAM/TSP patients, we examined TNF-α and IL-1β expression in cultured PBMCs of ND and HAM/TSP patients. After culture of total PBMCs for 24 hours, the frequency of CD14^+ ^cells that expressed TNF-α was first examined by flow cytometry. CD14^+ ^cells expressing TNF-α was significantly elevated in HAM/TSP patients, compared to NDs (Figure 2A). IL-1β was detected in PBMC culture supernatants of HAM/TSP patients but not of NDs (Figure 2B). Since relative expression of IL-1β mRNA dramatically increased in CD14^+ ^cells after culture (data not shown), IL-1β detected in the culture supernatants would be released from the MPs of HAM/TSP patients.

In addition to the production of various proinflammatory cytokines, activated or virus-infected MPs, such as infection with HIV, effectively transfer or promote productive virus upon interaction with T cells [30,31]. Although CD14^+ ^cells of patients with HAM/TSP are activated and also infected with HTLV-I at low levels, we wished to determine if there would be an increase in HTLV-I production in CD4^+ ^T cells of patients with HAM/TSP after interaction with autologous CD14^+ ^cells. To address whether CD14^+ ^cells promote HTLV-I production in CD4^+ ^T cells of patients with HAM/TSP, we examined HTLV-I Tax expression of CD4^+^CD25^+ ^T cells and CD4^+^CD25^- ^T cells cocultured with or without autologous CD14^+ ^cells of patients with HAM/TSP, compared to those of ACs. As shown in Figure 2Ci, in patients with HAM/TSP, 5.8-9.5% of CD4^+^CD25^+ ^T cells expressed HTLV-I Tax proteins at baseline. After coculture with autologous CD14^+ ^cells, HTLV-I Tax expression was dramatically increased in CD4^+^CD25^+ ^T cells (14.1-15.9%, p = 0.0226; Figure 2Ci). While HTLV-I Tax expression was also detected in 0.8-4.4% of CD4^+^CD25^- ^T cells, an increase after coculture with CD14^+ ^cells was lower than in CD4^+^CD25^+ ^T cells (Figure 2Cii). Since the increase of Tax expression was not detected in CD4^+ ^T cells without cell-cell contact with CD14^+ ^cells (data not shown), the increased expression of HTLV-I Tax in CD4^+ ^T cells by the addition of CD14^+ ^cells was cell-dependent. By contrast, both CD4^+^CD25^+ ^T cells and CD4^+^CD25^- ^T cells of ACs showed lower expression of Tax proteins (< 1%), which did not change after coculture with autologous CD14^+ ^cells (Figure 2C). Thus, CD14^+ ^cells could accelerate Tax expression in HTLV-I-infected CD4^+ ^T cells of patients with HAM/TSP.

### Minocycline inhibited MP activation and spontaneous lymphocyte proliferation of patients with HAM/TSP

**Figure 3:**
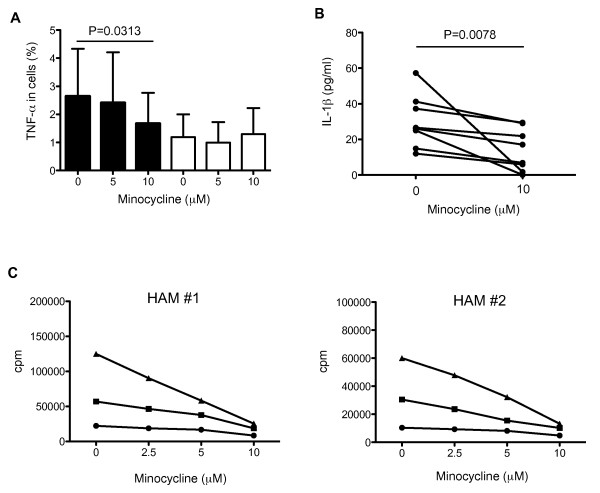
**Minocycline inhibited TNF-α expression and IL-1β release in patients with HAM/TSP**. (A) Dose-dependent inhibitory effects of minocycline on TNF-α expressions in CD14^+ ^cells (closed bar) and CD4^+ ^T cells (opened bar) of HAM/TSP patients (n = 6). The PBMCs were cultured with 0, 5 and 10 μM of minocycline for 24 hours. The frequency of CD14^+ ^cells expressing TNF-α was significantly inhibited at 10 μM of minocycline treatment in HAM/TSP patients (p = 0.0313; Wilcoxon matched-pairs signed rank test). Error bars represent SD. (B) Inhibition of IL-1β release in PBMC culture supernatants of HAM/TSP patients by 10 μM of minocycline after culture for 24 hours (n = 8). The release of IL-1β from these cultured HAM/TSP PBMCs was significantly inhibited by minocycline treatment (p = 0.0078; Wilcoxon matched-pairs signed rank test). (C) Inhibitory effects of minocycline on spontaneous lymphoproliferation in HAM/TSP patients. The PBMCs from two HAM/TSP patients (HAM#1 and #2) were cultured with 0, 2.5, 5 and 10 μM of minocycline, and pulsed with 1 μCi [^3^H] thymidine for 4 hours at 3 days (closed circle), 4 days (closed square) and 5 days (closed triangle). The average cpm from each well in triplicate was plotted.

Since various therapeutic agents have been developed for neuroinflammatory diseases specifically aimed at the inhibition of activated MPs, we attempted to examine the inhibition of MP function in patients with HAM/TSP using minocycline, which is known as an inhibitor of monocyte/macrophage activation. To evaluate inhibitory effect of minocycline on activated MP of patients with HAM/TSP, we examined TNF-α expression in cultured PBMCs of patients with HAM/TSP by treatment with minocycline. As shown in Figure 3A, the frequency of CD14^+ ^cells expressing TNF-α was significantly inhibited at 10 μM of minocycline treatment in HAM/TSP patients (Figure 3A; closed bar, p = 0.0313). The cultured CD4^+ ^T cells also expressed TNF-α, but minocycline did not inhibit TNF-α expression in CD4^+ ^T cells (Figure 3A; opened bar). As demonstrated previously (Figure 2B), IL-1β was detected in the supernatants of cultured PBMCs of patients with HAM/TSP; the release of IL-1β from these cultured HAM/TSP PBMCs was also inhibited by 10 μM of minocycline treatment (p = 0.0078; Figure 3B). These results demonstrated that minocycline inhibited the expression of proinflammatory cytokines from MPs, but not from CD4^+ ^T cells, of patients with HAM/TSP.

An additional established measure of HAM/TSP T cell activation *ex vivo *is the well-described observations of increased spontaneous lymphoproliferation [5]. In addition to the expression of HTLV-I Tax and a variety of cytokines in PBMCs of HTLV-I-infected patients that are associated with spontaneous lymphoproliferation [32-34], the activation of MP is also involved in spontaneous lymphoproliferation of patients with HAM/TSP [5]. To address the inhibitory effects of minocycline on spontaneous lymphoproliferation, uptake of [^3^H] thymidine as a marker of proliferation was examined in PBMCs of two patients with HAM/TSP after treatment with minocycline. In minocycline-treated HAM/TSP PBMCs, the spontaneous lymphoproliferation was inhibited in a dose-dependent manner (Figure 3C). Since the treatment with minocycline did not inhibit HTLV-I Tax expression in both T cells and CD14^+ ^cells (data not shown), these results showed that minocycline can downregulate MP activation, such as proinflammatory cytokine expression.

### Minocycline inhibits spontaneous degranulation and IFN-γ expression in CD8^+ ^T cell of patients with HAM/TSP

**Figure 4:**
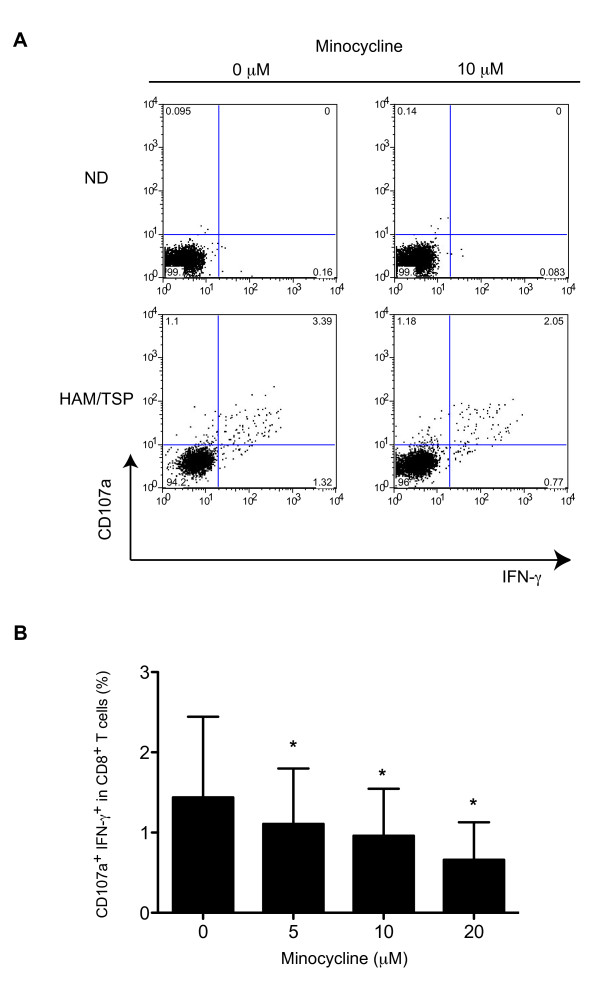
**Minocycline inhibited spontaneous degranulation/IFN-γ expression in CD8^+ ^T cells of patients with HAM/TSP**. (A) Representative dot plots of CD107a and IFN-γ expression in CD8^+ ^T cells of a ND and a HAM/TSP patient after culture for 24 hours with or without 10 μM of minocycline. (B) Inhibitory effects of minocycline on degranulation/IFN-γ expression in CD8^+ ^T cells of eight HAM/TSP patients after culture for 24 hours. Spontaneous degranulation/IFN-γ expression in CD8^+ ^T cells of HAM/TSP patients was significantly inhibited by minocycline treatment (*p = 0.0078; all by Wilcoxon matched-pairs signed rank test). Error bars represent SD.

MPs play an indispensable role in the induction of antigen-specific CTL responses by capturing viral antigen and presenting peptide through MHC class I to CD8^+ ^T cells. In patients with HAM/TSP, HTLV-I-infected or activated MPs collaborate with CD8^+ ^T cell to induce spontaneous degranulation and high IFN-γ expression [19]. Since we have demonstrated that minocycline has inhibitory effects on activated MPs (Figure 3), minocycline might also inhibit MP function such as triggering adaptive immune responses. To determine if inhibition of MPs affects CD8^+ ^T cell responses in HAM/TSP, we examined the effect of minocycline on expression of CD107a, a marker of degranulation, and IFN-γ in CD8^+ ^T cells of patients with HAM/TSP. As previously reported [19], CD107a and IFN-γ were spontaneously expressed in CD8^+ ^T cells of a patient with HAM/TSP after PBMC culture for 24 hours without any exogenous stimuli, but not in those cells of a ND. In Figure 4A, representative dot plots show that treatment with minocycline inhibited CD107a and IFN-γ expression in CD8^+ ^T cells of a patient with HAM/TSP. Group analysis showed significant, dose-dependent, inhibitory effects of minocycline on spontaneous degranulation and IFN-γ expression in CD8^+ ^T cells of patients with HAM/TSP (Figure 4B). These results demonstrated that spontaneous degranulation and IFN-γ expression in CD8^+ ^T cells of patients with HAM/TSP were inhibited by treatment with minocycline.

### Minocycline inhibits antigen-specific CD8^+ ^T cells responses in patients with HAM/TSP

**Figure 5:**
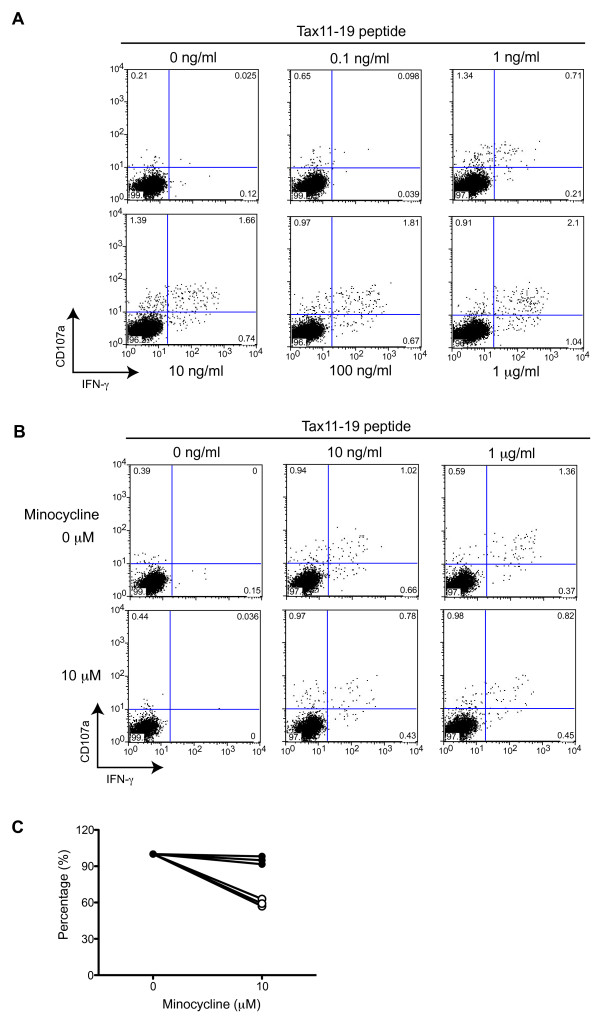
**Minocycline inhibited Tax11-19-specific IFN-γ expression in CD8^+ ^T cells of patients with HAM/TSP**. (A) Representative dot plots of CD107 and IFN-γ expression in CD8^+ ^T cells of a HLA-A*201^+ ^HAM/TSP patients, stimulated with Tax11-19 peptides. PBMCs were stimulated with Tax 11-19 peptide at concentration of 0, 0.1, 1, 10, 100 ng/ml, and 1 μg/ml for 5 hours. (B) Representative dot plots of Tax11-19 specific CD107 and IFN-γ expression in CD8^+ ^T cells of a HAM/TSP patient after treatment with or without 10 μM of minocycline. PBMCs were stimulated with Tax 11-19 peptide at concentration of 0, 10 ng/ml and 1 μg/ml for 5 hours. (C) Inhibitory effects of minocycline on IFN-γ expression, but not degranulation, in CD8^+ ^T cells of HAM/TSP patients after stimulation with 1 μg/ml of Tax11-19 peptides. The amounts of CD107a^+ ^(closed circles) and CD107a^+^IFN-γ^+ ^(opened circles) cells in CD8^+ ^T cells cultured without minocycline were normalized to 100%, and then, those in CD8^+ ^T cells cultured with minocycline were calculated. The graph was prepared from data obtained from three HLA-A*201^+ ^HAM/TSP patients. Tax11-19-specific IFN-γ expression, but not degranulation, in CD8^+ ^T cells of HAM/TSP patients was inhibited 40% by minocycline treatment.

To confirm whether treatment with minocycline could inhibit antigen-specific CD8^+ ^T cell responses of patients with HAM/TSP, we examined CD107a and IFN-γ expression in CD8^+ ^T cells of patients with HAM/TSP, who were subtyped as HLA-A*201, by stimulation with a known immunodominant HLA-A2-binding HTLV-I Tax11-19 peptide [35]. As previously reported [36], cytotoxicity (CD107 expression) can be triggered at peptide concentrations 10- to 100-fold less than those required for inflammatory cytokine (IFN-γ) production in primary virus-specific human CD8^+ ^T cells. In CD8^+ ^T cells of a patient with HAM/TSP, after stimulation with the low peptide concentration (0.1 ng/ml) for 5 hours, the majority of responding cells degranulated, but produced little or no detectable IFN-γ (Figure 5A). As the peptide concentration was increased, more cells exhibited dual effector functions of degranulation and IFN-γ production (Figure 5A). Thus, CD8^+ ^T cells exhibited inflammatory changes following cytotoxic responses depending on the quantity of antigen stimulation. Figure 5B shows representative dot plots of CD107a and IFN-γ expressions in CD8^+ ^T cells of a HLA-A*201^+ ^patient with HAM/TSP after the Tax11-19 stimulation with or without minocycline treatment. As the peptide concentration increased, more cells exhibited both degranulation and IFN-γ production in CD8^+ ^T cells of a HAM/TSP patient (Figure 5B, upper dot plots). Interestingly, as the cells were treated with minocycline, both degranulation and IFN-γ production were detected in Tax11-19-specific CD8^+ ^T cells, but the frequency of CD107a^+^IFN-γ^+ ^cell population did not increase in CD8^+ ^T cells stimulated with increased Tax11-19 peptides (Figure 5B, lower dot plots). These results suggested that minocycline inhibited the activation of Tax-specific CD8^+ ^T cells (Figure 5B, lower dot plots). In addition, IFN-γ expression was reduced, but total CD107a expression did not change in Tax11-19-specific CD8^+ ^T cells after minocycline treatment (Figure 5B, lower dot plots). Three HLA-A*201^+ ^HAM/TSP patients showed that minocycline treatment inhibited 40% of CD107a^+^IFN-γ^+ ^expressions, but not total CD107a expressions, in CD8^+ ^T cells after stimulation with Tax11-19 (Figure 5C). These results demonstrated that treatment with minocycline reduced the inflammatory responses (IFN-γ expression), but retained anti-viral cytotoxic response (total CD107a expression) in Tax11-19-specific CD8^+ ^T cells of HAM/TSP patients.

### Minocycline down-regulated MHC class I expression on MPs of patients with HAM/TSP

**Figure 6:**
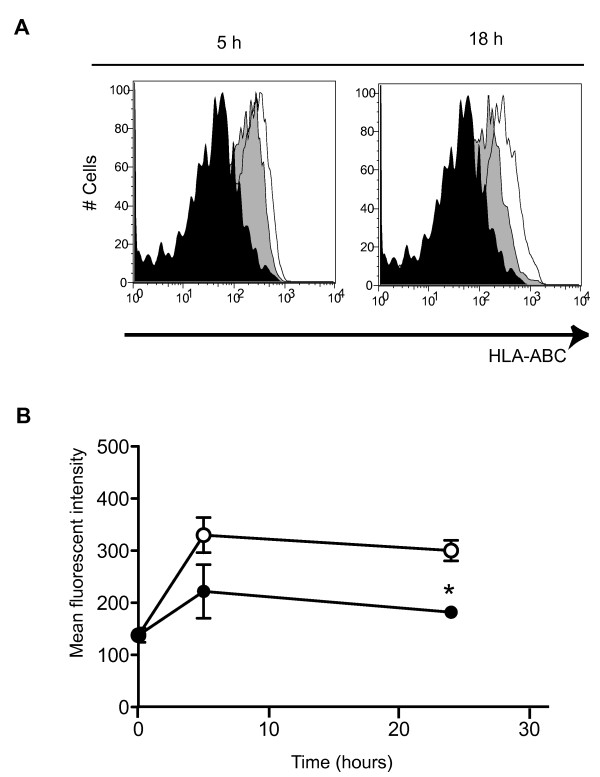
**Minocycline downregulated HLA-ABC expressions in CD14^+ ^cells of patients with HAM/TSP**. (A) Representative histograms of HLA-ABC expression on CD14^+ ^cells of a HAM/TSP patient. Staining on CD14^+ ^cells before culture (closed histogram) and after culture for 5 hours and 18 hours, with minocycline (grayed histogram) and without minocycline (opened histogram), were shown. (B) Comparison of HLA-ABC expression in CD14^+ ^cells of HAM/TSP patients after 18 hours culture with minocycline (closed circle) or without minocycline (opened circle). The mean fluorescent intensities of MHC class I expression on CD14^+ ^cells were significantly inhibited by treatment with minocycline at 18 hours culture (*p = 0.0382). Error bars represent SD.

As CD8^+ ^T cells are stimulated by antigenic peptides that are presented by MHC class I molecules expressed on the surface of antigen-presenting cells, we asked whether the effect of minocycline that modulates the inflammatory response in Tax-specific CD8^+ ^T cells of patients with HAM/TSP might be associated with decreased capacity of antigen-presentation in MPs. To clarify the capacity of antigen-presentation in MPs, we examined MHC class I expression on MPs of patients with HAM/TSP after culture with or without minocycline treatment. Figure 6A shows representative histograms of MHC class I expression on CD14^+ ^cells in a patient with HAM/TSP before and after culture for 5 and 18 hours. MHC class I expression on CD14^+ ^cells of a patient with HAM/TSP gradually increased after culture (Figure 6A). After treatment with minocycline, MHC class I expression on CD14^+ ^cells gradually decreased, compared to those on CD14^+ ^cells without minocycline (Figure 6A). Group analysis including three patients with HAM/TSP showed that mean fluorescent intensities of MHC class I expression on CD14^+ ^cells were significantly inhibited by treatment with minocycline after 18 hours culture (Figure 6B). These results demonstrated that minocycline down-modulated MHC class I expression on activated HAM/TSP MPs, suggesting that the inflammatory response of CD8^+ ^T cells in patients with HAM/TSP was suppressed through down-regulation of MP activation by minocycline.

## Discussion

MPs play pivotal roles in antigen capture and presentation, pathogen and tissue debris clearance, and cellular secretory functions. However, activated MPs can infiltrate through the blood brain barrier and contribute to the CNS inflammation by secreting various inflammatory cytokines and growth-inhibiting proteins. In HAM/TSP, MPs are reservoirs of HTLV-I, induce proinflammatory cytokines and excessive antigen-specific T cell responses, and can also infiltrate the CNS. In our study, we analyzed CD14^+ ^cell subpopulation in PBMCs of patients with HAM/TSP and demonstrated that CD14^low^CD16^+ ^subset of patients with HAM/TSP showed significantly higher CX_3_CR1 and HLA-DR expression, compared to NDs and ACs. Since it has been reported that CX_3_CR1 expression is regulated by IL-2 and IL-15 [37], activated T cells expressing these cytokines might affect CX_3_CR1 expression on monocytes in patients with HAM/TSP [19,38,39]. In mice, GR1^-^CX_3_CR1^high ^monocytes (homolog of human CD16^+ ^monocytes) patrol vascular endothelium by mechanisms involving LFA-1 and CX_3_CR1 and are rapidly recruited into inflamed tissues, such as spleen, gut, lung and brain, where they differentiate into macrophage [23,40]. In humans, CD16^+ ^monocytes that have the potential to migrate preferentially in response to fractalkine, a ligand of CX_3_CR1, have more Fc receptor mediated phagocytosis function and are at a more advanced stage of differentiation to macrophage and dendritic cell [41-43]. These findings suggest that CD14^low^CD16^+ ^and CD14^+^CD16^- ^cells are recruited into different anatomic sites under constitutive or inflammatory conditions and play distinct functional roles in immunity and disease pathogenesis. Fractalkine is expressed on activated endothelial cells [44], neuron [45], apoptotic cells [46], and brain with inflammation [47]. Therefore, HTLV-I-activated or infected cells might induce fractalkine expression at the site of inflammation such as the spinal cord to recruit and adhere CX_3_CR1^+ ^cells. The hypothesis was supported by the accumulation of CX_3_CR1^+ ^cells immunohistochemically detected in the meninges and parenchyma of HAM/TSP spinal cords as well as around blood vessels (Figure 1D). The CX_3_CR1^+ ^cells were CD68^+ ^and also morphologically consistent with MPs. Therefore, these results suggested that CX_3_CR1^+ ^MPs could accumulate in spinal cords of patients with HAM/TSP. Moreover, the increase of degranulation and IFN-γ expression in CD8^+ ^T cells were significantly correlated with the increase of CX_3_CR1 and HLA-DR expression in CD14^low^CD16^+ ^subset of HTLV-I-infected patients. These results support the hypothesis that strong correlation between CD8^+ ^T cell activation and MP activation contribute to the pathogenesis of HAM/TSP. These differential changes in peripheral MP subpopulations *in vivo *may also be associated with the infiltration of MPs into the CNS and CD8^+ ^T cell activation in patients with neurologic inflammatory disease.

MP activation in patients with HAM/TSP was also suggested by TNF-α and IL-1β expression in CD14^+ ^cells. Expression of IL-1β and TNF-α was detected in perivascular infiltrating macrophages and microglia in the spinal cords of patients with HAM/TSP and in infiltrating macrophage in the muscle of patients with HTLV-I-related myositis [27,48]. Thus, the proinflammatory cytokine expression in peripheral MPs might be related to the infiltration of MPs into the inflammatory site of patients with HTLV-I-related diseases. Moreover, CD14^+ ^cells accelerated HTLV-I Tax expression of autologous CD4^+^CD25^+ ^T cells in patients with HAM/TSP, which was dependent on cell-cell contact. In patients with HAM/TSP, high HTLV-I Tax expression is mainly detected in CD4^+ ^T cells after *ex vivo *culture, but dendritic cells and CD14^+ ^cells can also express HTLV-I Tax, consistent with the observation that HTLV-I infects dendritic cells to effectively transfer cell-free virus to CD4^+ ^T cells [18,19]. In HIV, human CD16^+ ^monocytes have been shown to be more susceptible to infection than CD16^- ^monocytes, to preferentially harbor the virus over the long-term, and to promote high levels of HIV replication upon differentiation into macrophages and interaction with activated T cells [30,49]. Therefore, HTLV-I infected and activated MP might likewise contribute to T cell activation and virus dissemination in HTLV-I associated disease.

Minocycline is a well known as inhibitor of MP activation and has been reported to have beneficial effects on inflammation, microglial activation, matrix metalloproteinases, nitric oxide production, and apoptotic cell death [29]. Furthermore, minocycline has been suggested to have neuroprotective effects in human as well as in animal models of a number of neurologic diseases including stroke, multiple sclerosis, and Parkinson's disease [29]. In our study, minocycline treatment significantly inhibited proinflammatory cytokine expression (TNF-α and IL-1β) in CD14^+ ^cells of patients with HAM/TSP, while TNF-α expressions in CD4^+ ^T cells of patients with HAM/TSP did not change. These results suggest that the effects of minocycline may act through inhibition of MP activation rather than HTLV-associated T cell activation. Unexpectedly, minocycline treatment also effectively inhibited spontaneous lymphoproliferation and IFN-γ expression of CD8^+ ^T cells, which are well-described observations of T cell activation in patients with HAM/TSP. While these T cell responses have been reported to be due to IL-2/IL-2 receptor and IL-15/IL-15 receptor autocrine loop following expression of HTLV-I Tax in T cells [32,38], a number of studies have demonstrated that non-T cells and CD14^+ ^cells can also play a stimulatory role in HTLV-I-associated T cell activation [5,19,38]. Therefore, our results support the view that T cell responses in patients with HAM/TSP are due, in part, to the activation of MPs.

Inhibition of MPs resulted in the suppression of CD8^+ ^T cell dysregulation (degranulation and IFN-γ expression). Elevated IFN-γ expression is an important immunological marker in the pathogenesis of HAM/TSP [50], and CD8^+ ^T cell dysregulation was mediated by various factors, including virus infection, enhanced IL-2/IL-15, and expression of cellular molecules [19,51-54]. Unexpectedly, minocycline inhibited spontaneous degranulation/IFN-γ expression in CD8^+ ^T cells of HAM/TSP patients as well as HTLV-I Tax11-19-specific CD8^+ ^T cell responses. Antiviral CD8^+ ^T cells can elaborate at least two effector functions, cytotoxicity and inflammatory cytokine production, which are determined primarily by antigen concentration [36]. Interestingly, minocycline treatment suppressed inflammatory IFN-γ production, but not total cytotoxicity (CD107a expression) in Tax-specific CD8^+ ^T cells of patients with HAM/TSP. Moreover, after the treatment with minocycline, MHC class I expression on CD14^+ ^cells of patients with HAM/TSP was gradually suppressed in cultured cells, compared to untreated MPs. These results suggested that the activation of CD8^+ ^T cells was inhibited through MHC class I downregulation on CD14^+ ^cells after minocycline treatment. This may be one mechanism involved in the reduction of CD8^+ ^T cell inflammatory IFN-γ production in the presence of minocycline. Moreover, minocycline significantly inhibited spontaneous degranulation/IFN-γ expression in CD8^+ ^T cells of HAM/TSP patients. As previously reported, the spontaneous degranulation/IFN-γ expression in CD8^+ ^T cells of HAM/TSP patients was mediated by various factor(s) [19,52]. To evaluate regulatory effects of CD8^+ ^T cell by minocycline, further analysis would be needed. In addition, even though minocycline down-modulates the capacity of antigen-presenting cells to trigger CD8^+ ^T cell effector responses, the cytotoxic function of Tax-specific CD8^+ ^T cells might be still maintained and continue to provide control of virus-infected cells. This may have a positive clinical consequence for use of minocycline in treatment of HTLV-I-associated disease.

## Conclusions

Collectively, these results suggest that minocycline does not only inhibit the activation of MPs of patients with HAM/TSP, but also HTLV-I-associated T cell activation such as lymphoproliferation and inflammatory cytokine production of CD8^+ ^T cells through the downregulation of MP function. Thus, the inhibition of HTLV-I-infected or activated MPs may be of clinical use in the treatment of patients with HTLV-I-associated neurological disease.

## Methods

### Patient samples

Blood samples were obtained from twelve patients with HAM/TSP (HAM#1-12), six HTLV-I-seropositive asymptomatic carriers (AC#1-6), and ten HTLV-I-seronegative healthy donors (ND#1-10). Diagnosis of HAM/TSP was based on WHO diagnostic criteria. Three patients with HAM/TSP were HLA-A*201^+^. PBMCs were isolated by Ficoll-Hypaque (Lonza Walkersville, Walkersville, MD) centrifugation. The PBMCs obtained from HTLV-I-infected patients or ND were cryopreserved in liquid nitrogen until use. Informed consent was obtained from each subject. The study was reviewed and approved by the National Institute of Neurological Disorders and Stroke (NINDS) Institutional Review Board. Informed consent was obtained in accordance with the Declaration of Helsinki.

### Antibodies and reagents

For flow cytometry, antibodies for human CD3, CD4, CD8, CD14, CD16, CD28, CD49d, CD107a, IFN-γ, TNF-α, and HLA-DR (all from BD Biosciences, San Jose, CA), CX_3_CR1 (Medical and Biological laboratories, Nagoya, Japan), HLA-ABC (AbD Serotec, Oxford, UK), and anti-Tax monoclonal antibody (Lt-4) were used. For immunohistochemistry, rabbit polyclonal anti-human CX_3_CR1 (abcam, Cambridge, MA) was used as primary antibody. Minocycline was purchased from Sigma (St. Louis, MO).

### Cell culture

PBMCs of NDs or patients with HAM/TSP were suspended at 1 × 10^6 ^cells/mL in RPMI media (RPMI 1640 supplemented with 10% heat-inactivated fetal bovine serum, 100 U/mL penicillin, 100 μg/mL streptomycin sulfate, and 2 mM L-glutamine), and cultured for 24 hours either with or without minocycline in 24 well plate in 5% CO_2 _incubator at 37°C. The culture supernatants were collected, centrifuged at 2000 g for 10 min to remove cellular debris and stored at -80°C until use. The cultured cells were collected for immunofluorescence staining or stored at -80°C until use. For immunofluorescence staining of MHC class I on MPs, PBMCs were collected after culture for 5 hours or 18 hours.

To examine Tax expression in CD4^+ ^T cells cocultured with or without CD14^+ ^cells, CD4^+^CD25^+ ^T cells or CD4^+^CD25^- ^T cells and CD14^+ ^cells were magnetically isolated from PBMCs of HTLV-I-infected patients by CD4^+^CD25^+ ^Regulatory T cell Isolation Kit and CD14 MicroBeads (both from Miltenyi Biotec, Bergisch Gladbach, Germany), respectively, according to the manufacture's instruction, and 2 × 10^5 ^cells of each CD4^+ ^T cells were cocultured with or without the same amount of autologous CD14^+ ^cells for 18 hours in 48 well plate in 5% CO_2 _incubator at 37°C.

### ELISA

IL-1β was detected in the PBMC culture supernatants of NDs and patients with HAM/TSP using Human IL-1β Quantikine ELISA (R & D systems), according to the manufacturer's instructions

### CD107a mobilization assay

CD107a mobilization assay was performed as previously described [19]. To detect spontaneous degranulation and IFN-γ expression in CD8^+ ^T cells, PBMCs of patients with HAM/TSP were cultured for 24 hours. To detect Tax11-19 specific responses, PBMCs were stimulated with an appropriate concentration of HTLV-I Tax11-19 LLFGYPVYV and 1 μg/mL each of CD28 and CD49d for 5 hours. In treatment of minocycline, appropriate minocycline was added into the culture. Conjugated CD107a antibody, 0.7 μL/mL of GoldiStop™ (BD Biosciences), and 1 μg/mL of brefeldin A (Sigma) were added into the culture for 5 hours before the time point for detection.

### Flow cytometry

For analysis of peripheral blood monocyte populations, patients' PBMCs were stained with CD3, CD4, CD8, CD14, CD16, HLA-DR and CX_3_CR1. Expression of CD107a, IFN-γ, TNF-α and intracellular Tax in the cultured or uncultured PBMCs was examined by flow cytometoric analysis. First, PBMCs were surface-stained with specific antibodies. After fixation and permeabilization with Fixation/Permeabilization solution (BD Biosciences) according to the manufacturer's instructions, the cells were intracellularly stained with anti-IFN-γ, anti-TNF-α or anti-Tax for each experiment. Flow cytometric analysis was performed using a FACSCalibur flow cytometer (BD Biosciences) or LSR II (BD Biosciences). The data were analyzed using FlowJo software (Tree Star, San Carlos, CA).

### Lymphoproliferation assay

Lymphoproliferation assay was performed as previously described [55]. PBMCs were suspended in RPMI medium supplemented with 5% human AB serum, 100 U/mL penicillin, 100 μg/mL streptomycin sulfate, and 2 mM L-glutamine, and plated in triplicate on a round bottom 96-well plate at a concentration of 3 × 10^5 ^cells/well with or without minocycline. The cells were cultured in 5% CO_2 _incubator at 37°C, and pulsed after 3 to 5 days of culture for 4 h with 1 μCi [^3^H] thymidine. The average cpm from each of the wells was plotted.

### Immunohistochemistry

Spinal cord tissues from a patient with HAM/TSP were fixed with buffered formalin and embedded in paraffin wax. Microtome sections were cut 10 μm thick. Sections were deparaffinized with xylene, rehydrated and immersed in Target Retrieval Solution, pH6.0, (Dako, Carpinteria, CA) at 121°C for 10 min. After blocking of endogenous peroxide with 3% hydrogen peroxide for 10 min, the sections were incubated with a rabbit anti- CX_3_CR1 antibody (1 μg/ml) for one hour at room temperature. Reactivity was visualized with diaminobenzidine (DAB) using Envision™+system (Dako), followed by counterstaining with hematoxylin. The stained sections were visualized with Zeiss 200M Axiovert inverted microscope (Carl Zeiss MicroImaging Inc, Thornwood, NY). The image data of each section were created using Volocity imaging analysis software (Improvision, Waltham, MA).

### Statistical analysis

Mann-Whitney test and Wilcoxon matched-pairs signed rank test were used for comparison between groups. Simple linear regression analysis was used for explaining a relationship between groups, respectively. All statistical analysis was performed using Prism (GraphPad software).

## Competing interests

The authors declare that they have no competing interests.

## Authors' contributions

YE-A designed the research, performed most of the experiments, analyzed results, made the figures and wrote the manuscript; EM analyzed immunohistochemical image, analyzed results, made the figures and wrote the manuscript; UO coordinated clinical work, analyzed results and wrote the manuscript; YT contributed reagents for analysis. SJ designed the research, analyzed results and wrote the manuscript. All authors read and approved the final manuscript.
